# Metabolic Flux Analysis Reveals Cell Line‐Specific Rewiring in CHO Cells Following TCA Cycle Intermediate Feeding for Bioprocess pH Control

**DOI:** 10.1002/bit.70287

**Published:** 2026-06-28

**Authors:** Sarah A. Sacco, Kevin J. Ruiz‐Márquez, Rachel Moen, Irina Trenary, Xiaolin Zhang, Xiaowen Wang, Malik Padellan, Henry Lin, Jamey D. Young

**Affiliations:** ^1^ Department of Chemical and Biomolecular Engineering Vanderbilt University Nashville Tennessee USA; ^2^ Biologics Process Research & Development, Process Research & Development Merck & Co. Inc. Rahway New Jersey USA; ^3^ Department of Molecular Physiology and Biophysics Vanderbilt University Nashville Tennessee USA

## Abstract

Chinese hamster ovary (CHO) cells are the leading host for recombinant therapeutic protein production in the biopharma industry. In this study, we investigated how feeding acidic forms of tricarboxylic acid (TCA) cycle intermediates—malic acid, succinic acid, and α‐ketoglutaric acid—affects cell culture performance and metabolism in two industrial IgG‐producing CHO cell lines. These intermediates were used as pH control agents to replace conventional CO_2_ sparging, enabling simultaneous modulation of the bioreactor environment and cell metabolism. Carbon‐13 metabolic flux analysis (^13^C‐MFA) revealed substantial rewiring of central carbon and nitrogen metabolism under all fed conditions, with distinct responses between cell lines. Intermediate‐fed cultures exhibited enhanced TCA cycle fluxes, reduced glucose dependency, decreased lactate accumulation, and altered routing of pyruvate. Notably, α‐ketoglutaric (α‐KG) acid feeding triggered divergent nitrogen assimilation phenotypes: one cell line enhanced glutamine biosynthesis and ammonium clearance, while the other accumulated glutamate with minimal glutamine production. These metabolic adaptations were accompanied by shifts in redox balance and delayed but measurable increases in cell‐specific IgG productivity. Our findings highlight the compound‐ and cell line‐specific nature of metabolic responses to TCA cycle intermediate feeding and support its use for pH control and bioprocess optimization.

## 1 Introduction

The therapeutic protein market has experienced remarkable growth since the introduction of the first commercially available recombinant DNA product over 35 years ago. In 2021, the industry reached a significant milestone with the approval of the 100th antibody by the FDA, which reflects the increasing popularity of therapeutic protein products with newer modalities entering diverse fields for diagnostics and treatments. However, meeting the growing demand for protein therapeutics remains a major challenge in the industry. This challenge has driven innovation to improve productivity, reduce manufacturing cost, and achieve shorter timelines at each stage of the protein therapeutic production process, while maintaining stable product quality throughout these advancements.

Chinese hamster ovary (CHO) cells are widely recognized as the “workhorse” of the biopharmaceutical industry, being employed in nearly 70% of all protein therapeutic production processes (De Jesus and Wurm 2011; Wurm 2004). The popularity of CHO cells stems from their ability to perform human‐like post‐translational modifications, grow in suspension culture, and survive in chemically defined, serum‐free media (Majewska et al. 2020; Rodrigues et al. 2013; Fischer et al. 2015). From an upstream process perspective, media and process optimization have led to higher viable cell density (VCD) and/or improved volumetric titer, with final titers routinely reaching 10 g/L (Jayapal et al. 2007). However, from a cell biology standpoint, achieving such high levels of productivity places significant energetic demands on CHO cells, as formation of each peptide bond requires four ATP equivalents, and a typical monoclonal antibody (mAb) can contain over 600 individual amino acids (Seth et al. 2006).

Previous studies have indicated that high‐producing CHO cell lines often exhibit upregulated tricarboxylic acid (TCA) cycle flux, likely to meet the high energy requirements of protein biosynthesis (Park et al. 2022; Templeton et al. 2017a; Sacco et al. 2023). Glucose is the primary carbon source for CHO cell cultures and is metabolized through glycolysis and the TCA cycle to provide energy and biosynthetic precursors for cell growth and recombinant protein production. Lactate, a major metabolic byproduct that accumulates in the culture media, serves as an indirect indicator of the flux balance between glycolysis and the TCA cycle. Lactate is typically produced during periods of high glycolytic flux (such as the exponential growth phase) and consumed during periods of elevated TCA cycle flux (such as the stationary phase) (Ahn and Antoniewicz 2013). It has been hypothesized that this transition from lactate production to consumption, known as the “lactate switch,” helps maintain cellular redox balance (Hartley et al. 2018) and is typically a hallmark of high‐producing cell cultures (Le et al. 2012). However, excessive lactate production can lead to low pH (if it is uncontrolled) or high osmolality when base addition is used for pH control (Gagnon et al. 2011). While lactate can serve as a secondary carbon source to fuel the TCA cycle during the stationary phase (Templeton et al. 2014), there are inherent drawbacks associated with high extracellular lactate concentrations (Pereira et al. 2018).

Process conditions, such as culture pH, temperature, and dissolved oxygen, are known to influence lactate metabolism. Media additions, such as increasing copper concentration or supplementing the culture with slowly metabolizable carbon sources, can also play a critical role in enhancing lactate consumption (Gagnon et al. 2011; Altamirano et al. 2006, 2000). Moreover, TCA cycle intermediates have been supplemented to promote lactate consumption. Previous research by Zhang et al. (2020) characterized the effects of adding TCA cycle intermediates in the basal media or feed solution. The study found that adding TCA cycle intermediates to the basal media did not affect culture growth and had no detrimental effects on culture performance even at higher concentrations. Feeding α‐ketoglutaric (α‐KG) acid, malic acid, and/or succinic acid in the stationary phase significantly improved lactate consumption, reduced ammonium accumulation, and led to higher cell‐specific productivity (qP) and antibody titer. However, the underlying metabolic changes that led to this improved culture performance were unclear.

The findings presented in this study build upon the previous work by Zhang et al. (2020). While their prior study demonstrated that supplementation with TCA cycle intermediates could improve culture productivity, it did not address the intracellular basis for these improvements, including the metabolic fate of the supplemented intermediates or the mechanisms underlying the increase in qP. The objective of the present study was therefore to determine how using malic acid, succinic acid, and α‐ketoglutaric acid as alternative pH‐control agents influences intracellular metabolism in mAb‐producing CHO cells, and to clarify how carbon supplied by these intermediates is redistributed through central carbon metabolism under fed‐batch conditions. Herein, carbon‐13 metabolic flux analysis (^13^C‐MFA) was carried out on two mAb‐expressing CHO cell lines (denoted A and B) in fed‐batch bioreactor cultures that utilized the TCA cycle intermediates malic acid, succinic acid, or α‐KG acid for pH control in place of CO_2_ sparging. Both cell lines exhibited a lactate switch; however, their lactate profiles diverged during the stationary phase. In Cell Line B, lactate was consumed throughout stationary phase under all feeding conditions, whereas in Cell Line A, lactate was produced again during late stationary phase—specifically during the isotope labeling study—by CO_2_‐sparged control cultures but not TCA cycle intermediate‐fed cultures. To rule out any effects of CO_2_ on lactate metabolism, hydrochloric (HCl) acid was also examined as an alternative pH control condition in cultures of Cell Line B, allowing for a clearer analysis of the lactate profile and TCA cycle activity resulting from intermediate feeding while controlling for differences in CO_2_ sparging. By comparing the distinct metabolic profiles of the two cell lines and using the additional HCl control condition for Cell Line B, the study provided a deeper understanding of how TCA cycle intermediate supplementation affects cultures that either consume or produce lactate in the stationary phase. Additionally, a detailed map of the metabolic alterations resulting from feeding TCA cycle intermediates was obtained.

## 2 Materials and Methods

### 2.1 Cell Culture

Two in‐house IgG1‐expressing CHO cell lines, designated as Cell Lines A and B, developed by Merck & Co. Inc. (Rahway, NJ, USA) using glutamine synthetase (GS) selection, were used for all of the bioreactor experiments in this study, and culture conditions were as previously reported (Zhang et al. 2020). In brief, cell bank vials were thawed and expanded in shake flasks maintained at 5% CO_2_ and 36.5°C in humidified bioreactors. For Cell Line A, a total of eight independently controlled ambr250 bioreactors (Sartorius Stedim, Göttingen, Germany) were inoculated to a target cell density of 2.0 × 10^6^ cells/mL in a glucose‐containing proprietary basal media. A glucose‐containing feed was added once daily at amounts determined based on the residual glucose concentration. Glutamic acid was fed as a bolus on days when the measured glutamate concentration fell below 3 mM, restoring the concentration to 5 mM after feeding. Dissolved oxygen (DO) was maintained at 30% air saturation by sparging O_2_. The pitched blade impellers in each bioreactor were set to an initial rate of 400 rpm; once the O_2_ sparge rate reached 5 mL/min, the agitation speed was increased as a secondary control element for DO. Temperature was maintained at 36.5°C. For foam control, EX‐CELL antifoam (Sigma‐Aldrich, St. Louis, MO) was added as needed.

For pH control, CO_2_ was sparged as needed for the first 6 days of culture for all bioreactors to maintain a pH of 6.90 with a deadband of 0.05. On day 6, control bioreactors continued to receive CO_2_ for pH control while the TCA cycle intermediate‐fed bioreactors began to receive either malic acid, succinic acid, or α‐KG acid as needed to control pH. For Cell Line B, a similar experimental strategy was followed using eight bioreactors. Two separate bioreactors were additionally included to investigate the use of HCl acid for pH control beginning on day 6. This additional condition was designed to assess potential confounding effects associated with changes in dissolved CO_2_ on culture performance (Brunner et al. 2017), thus providing further context for the interpretation of experimental outcomes, especially regarding the impact of TCA cycle intermediate supplementation. Because the pH of all bioreactors was controlled by CO_2_ sparging prior to day 6, any differences in culture performance observed before day 6 are attributable to baseline variation rather than treatment effects. Offline pH and pCO_2_ profiles from day 6 to the end of culture are provided in Supporting Information Figure S1.

### 2.2 Isotope Labeling Experiment (ILE)

The isotope labeling experiment was carried out on‐site at Merck & Co. Inc. (Rahway, NJ, USA). Two time periods of interest were studied: days 10–12 (stationary phase) for Cell Line A, and days 9–11 (stationary phase) for Cell Line B. The isotope labeling window was shifted earlier by 1 day for Cell Line B, due to its faster lactate consumption rate, to avoid the possibility of lactate becoming fully depleted before the end of the study. For each time period, a set of bioreactors was used to compare CO_2_ or HCl control conditions with the effects of feeding TCA cycle intermediates. At the beginning of stationary phase (day 9 or 10), a bolus of [U‐^13^C_6_] glucose (Cambridge Isotope Laboratories Inc.) was added to each bioreactor, resulting in glucose concentrations of 7–10 g/L (Supporting Information Figure S2). This final concentration was calculated to ensure glucose would not become fully depleted before the end of the labeling study. The feeding and sampling schemes are shown in Tables 1 and 2, respectively.

**Table 1:** Example feeding plan for each bioreactor during the time period of labeling.

T = 0 h	T = 24 h	T = 48 h
Proprietary feed solution	Proprietary feed solution	Quench and harvest remainder of culture
Glutamic acid	Glutamic acid	
6 g/L [U‐^13^C_6_] glucose	No glucose feed	

*Note:* The tracer ([U‐^13^C_6_] glucose) was added at T = 0 h along with a proprietary amino acid feed solution and glutamic acid. Twenty‐four hours after the tracer was added, the proprietary feed solution and glutamic acid were added to a set target concentration. Forty‐eight hours after adding the tracer, the remaining culture was quenched and harvested.

**Table 2:** Sampling plan for ILE. Samples were taken just before adding tracer and at 24, 36, and 48 h after tracer addition.

Before adding tracer (T = 0 h)	Quenched cell pellet, spent media
After adding tracer (T = 24 h)	Quenched cell pellet, spent media
T = 36 h	Quenched cell pellet, spent media
T = 48 h	Quenched cell pellet, spent media

Samples were collected just before the tracer was added and again at 24, 36, and 48 h after tracer addition. For spent media samples, 1 mL of culture was collected from each bioreactor, centrifuged to remove cells, and the supernatant was stored at −80°C. Cell pellets were cold‐quenched as previously described (Templeton et al. 2013). In brief, a quenching solution consisting of 60% methanol and 40% ammonium bicarbonate (0.85% w/v solution in water) was pre‐chilled to at least −40°C in a 4.5 M CaCl_2_ bath. Based on VCD measurements, a volume of cell culture containing 5 × 10^6^ viable cells was calculated and an amount of quenching solution equal to 5‐times the volume of cell culture was aliquoted for each sample. The required volume of cell culture was removed and immediately mixed with the aliquoted quenching solution. Samples were then centrifuged at 1000 RCF at 0°C for 1 min and cell pellets were stored at −80°C for further analysis.

### 2.3 Extraction and Derivatization of Intracellular Metabolites

Metabolites were extracted from the quenched cell pellets and derivatized as previously described (Templeton et al. 2013). In brief, each cell pellet was resuspended in a mixture of 4 mL of −20°C chloroform and 2 mL of −20°C methanol. The resuspension was then vortexed at 4°C for 30 min. Next, 1.5 mL of ice‐cold water and 6 µL of a 10 mM norvaline internal standard were added, and samples were vortexed for another 5 min at 4°C. Samples were then centrifuged at 4000 RCF for 20 min at 4°C. After centrifugation, the top, aqueous phase was collected and allowed to dry under air flow overnight. The dried sample was then resuspended in 50 µL of methoxyamine (MOX) reagent (Pierce; Rockford, IL). The resuspension was sonicated for 30 min at room temperature and then incubated at 40°C for 90 min. Next, 70 µL of MTBSTFA + 1% TBDMCS in pyridine (Pierce; Rockford, IL) was added, and the sample was incubated at 70°C for 30 min to convert metabolites to their respective *tert*‐butyldimethylsilane (TBDMS) derivatives. Finally, the sample was centrifuged at 14,000 rpm for 10 min and transferred to a vial before analysis by gas chromatography‐mass spectrometry (GC‐MS).

### 2.4 Extraction and Derivatization of Metabolites From Spent Media

For analysis of the glucose tracer enrichment in the media, glucose was converted to its di‐*O*‐isopropylidene propionate (DIO) derivative as described previously (Sacco et al. 2022). In brief, 20 µL of media was mixed with 300 µL of ice‐cold acetone. After centrifugation to precipitate protein, the supernatant was transferred to a glass test tube and dried under air flow at 60°C. The dried sample was resuspended in 500 µL of a 1:46 v/v mixture of 96% sulfuric acid and acetone, then incubated at room temperature for 60 min. The reaction was neutralized with 400 µL of 0.44‐mM sodium carbonate, then mixed with 1.5 mL of saturated sodium chloride solution and 1 mL of ethyl acetate. Tubes were vortexed and allowed to incubate at room temperature until the phases were completely separated. The upper organic layer was transferred to a microcentrifuge tube and evaporated to dryness under air flow at room temperature. The dried sample was resuspended in 150 µL of a 2:1 propionic anhydride and pyridine mixture and incubated for 30 min at 60°C. Samples were evaporated to dryness under air flow at 60°C. The dried sample was redissolved in 100 µL ethyl acetate, transferred to a glass GC‐MS vial insert, and analyzed using GC‐MS.

To measure the residual concentrations of TCA cycle intermediates, spent media samples were extracted and derivatized alongside standard curves containing the target metabolites at known concentrations. For the extraction, 30 µL of either sample or standard was mixed with 360 µL of a 2:1 mixture of ice‐cold methanol and chloroform. This mixture was vortexed at 4°C for 10 min. Next, 120 µL of ice‐cold chloroform, 210 µL of ice‐cold water, and 6 µL of a 10‐mM norvaline internal standard were added to each sample. Samples were vortexed to ensure they were well‐mixed, then centrifuged at 14,000 rpm at 0°C for 10 min. The aqueous phase was transferred to a new microcentrifuge tube, and the sample was dried under air flow overnight at room temperature. The dried sample was then derivatized with MOX and TBDMS reagents as described in Section 2.3.

### 2.5 Gas Chromatography‐Mass Spectrometry (GC‐MS)

Derivatized extracts were injected into an Agilent 7890 A gas chromatograph with an Agilent HP‐5ms column (30 m × 0.25 mm i.d. × 0.25 µm) connected to an Agilent 5977B mass spectrometer. As described previously (Sacco et al. 2022), TBDMS‐derivatized cell extract samples were eluted using the following temperature program: held at 80°C for 1 min, increased to 140°C at a rate of 20°C/min, then increased from 140°C to 234°C at 4°C/min, held at 234°C for 5 min, and finally increased from 234°C to 285°C at a rate of 20°C/min. A sample volume of 1 µL was injected at a 5:1 split ratio and a column flow rate of 1 mL/min. The gain factor was dynamically adjusted using the timed event mode to achieve a linear detector response over a wide range of metabolite concentrations; lower sensitivity was applied to detect more abundant metabolites while higher sensitivity was used for less abundant metabolites. The same parameters were used to run TBDMS‐derivatized media samples, but with a 15:1 split ratio. DIO‐derivatized media samples were eluted using the following temperature program: held at 80°C for 1 min, increased to 220°C at a rate of 40°C/min, then increased to 240°C at a rate of 10°C/min. A sample volume of 1 µL was injected at a 30:1 split ratio and a column flow rate of 1 mL/min.

### 2.6 Determination of Growth and Extracellular Flux Rates

VCD and viability were measured on a Cedex Hi‐Res cell counter (Roche; Mannheim, Germany) using the trypan blue exclusion method to determine viability. Culture pH, pO_2_, and pCO_2_ were measured offline using an ABL80 blood gas analyzer (Radiometer; Copenhagen, Denmark). Glucose, lactate, glutamate, glutamine, and ammonium were measured daily before any feed additions using a BioProfile FLEX2 (Nova Biomedical; Waltham, MA). Antibody titer was measured using an Agilent 1100 high‐performance liquid chromatography (HPLC) with a Protein A column (Agilent Technologies; Santa Clara, CA). All titer measurements were normalized to the peak titer in the control bioreactor.

Specific growth rate and cell‐specific consumption and production rates for unfed metabolites were determined using the ETA software package (Murphy and Young 2013), as previously described (Templeton et al. 2013). For metabolites that were fed during the ILE, cell‐specific rates were calculated using Equation 1.1:

(1.1) $$q_{i}=\frac{\left(C_{i}\cdot V_{i}\right)-\left(C_{i-1}\cdot V_{i-1}\right)-\sum_{j}C_{f}{\cdot V}_{f,j}\;}{X_{i}-X_{i-1}}$$

where *q*_*i*_ is the cell‐specific production rate at time *t*_*i*_, *C*_*i*_ is the metabolite's concentration in the bioreactor at time *t*_*i*_, *V*_*i*_ is the working volume of the bioreactor at time *t*_*i*_, *C*_*f*_ is the metabolite's concentration in the feed, *V*_*f,j*_ is the volume fed at time *t*_*j*_ (where *t*_*i*_ *_− _*_1_ ≤ *t*_*j*_ ≤ *t*_*i*_), and *X*_*i*_ is the integrated viable cell count (IVCC) at time *t*_*i*_.

For mAb production, the qP was determined using Equation 1.2:

(1.2) $${\mathrm{qP}}_{i}=\frac{\left({\mathrm{mAb}}_{i}\cdot V_{i}\right)-\left({\mathrm{mAb}}_{i-1}\cdot V_{i-1}\right)\;}{X_{i}-X_{i-1}}$$

where *qP*_*i*_ is the mAb specific productivity at time *t*_*i*_, and *mAb*_*i*_ is the mAb titer at time *t*_*i*_.

### 2.7 Determination of Extracellular and Intracellular Metabolite Concentrations and Redox Markers

The PIRAMID software package (publicly available at https://mfa.vueinnovations.com) was used for the quantification and analysis of MS datasets obtained from stable ILEs of Cell Lines A and B (Gomez et al. 2023). Total ion counts (TICs) for all metabolites measured in the TBDMS‐derivatized samples and standards were normalized to the TIC of norvaline *m/z* 288, a fragment ion of the internal standard added during extraction. Using the standard curves run alongside the samples, the absolute amounts of all metabolites of interest in the media and in the cell pellets were determined. For extracellular metabolites, this amount was normalized to the volume of media extracted to determine absolute concentration. For intracellular metabolites, the amounts were normalized to total viable cell counts. As described previously (Rahim et al. 2022; Krebs and Veech 1969), the cytosolic NADH/NAD^+^ ratio was estimated based on the equilibrium of lactate dehydrogenase (LDH) using Equation 2.1:

(2.1) $$\frac{\mathrm{NADH}}{{\mathrm{NAD}}^{+}}=\frac{\left[\mathrm{Lactate}\right]\times K_{\mathrm{LDH}}\;}{\left[\mathrm{Pyruvate}\right]}$$

where the concentrations of lactate and pyruvate were determined as described above and *K*_LDH_ = 1.11 × 10^−4^.

### 2.8 Carbon‐13 Metabolic Flux Analysis (^13^C‐MFA)

A previously published model of CHO cell metabolism was used for ^13^C‐MFA in this study (Templeton et al. 2017a). The model is a compartmentalized representation of central carbon metabolism and contains equations for reactions in glycolysis, pentose phosphate pathway (PPP), TCA cycle, amino acid catabolism, cell growth, and IgG biosynthesis (84 reactions). Exchange reactions between the media and cytosol for α‐KG, malate, and succinate were included in the model to account for uptake of the fed TCA cycle intermediates. Coefficients for the cell growth equation were based on a previously published study using the same parental cell line (Templeton et al. 2017b).

The INCA software package (Young 2014) (publicly available at https://mfa.vueinnovations.com) was used to fit the experimental measurements to the metabolic model, as previously described (Templeton et al. 2017b). Isotopic steady state was not achieved for all measured metabolites; therefore, isotopically nonstationary MFA (INST‐MFA) was utilized to calculate fluxes (Pereira et al. 2018). Fluxes were estimated using the Levenberg–Marquardt optimization algorithm. A minimum of 50 restarts from random initial guesses was used for each flux estimation to ensure a global minimum was found. To assess goodness‐of‐fit, flux results were subjected to a Chi‐square statistical test; all results are reported in Supporting Information Table S1. Additionally, 95% confidence intervals were calculated for each best‐fit flux value using INCA's parameter continuation function.

### 2.9 Statistical Analysis

One‐way and two‐way ANOVA were used to determine statistical significance between different feeding conditions, with *α* = 0.05. A Tukey multiple comparison test was applied if differences were detected. The standard error of the mean (SEM) was estimated for intracellular fluxes using the formula SEM = (UB − LB)/3.92, where UB and LB represent the upper and lower bounds of the calculated 95% confidence intervals and 3.92 is the number of standard errors that span the 95% confidence interval of a normally distributed random variable.

## 3 Results

### 3.1 TCA Cycle Intermediate Supplementation Had Cell‐Specific Effects on Growth and qP During Stationary Phase

**Figure 1:**
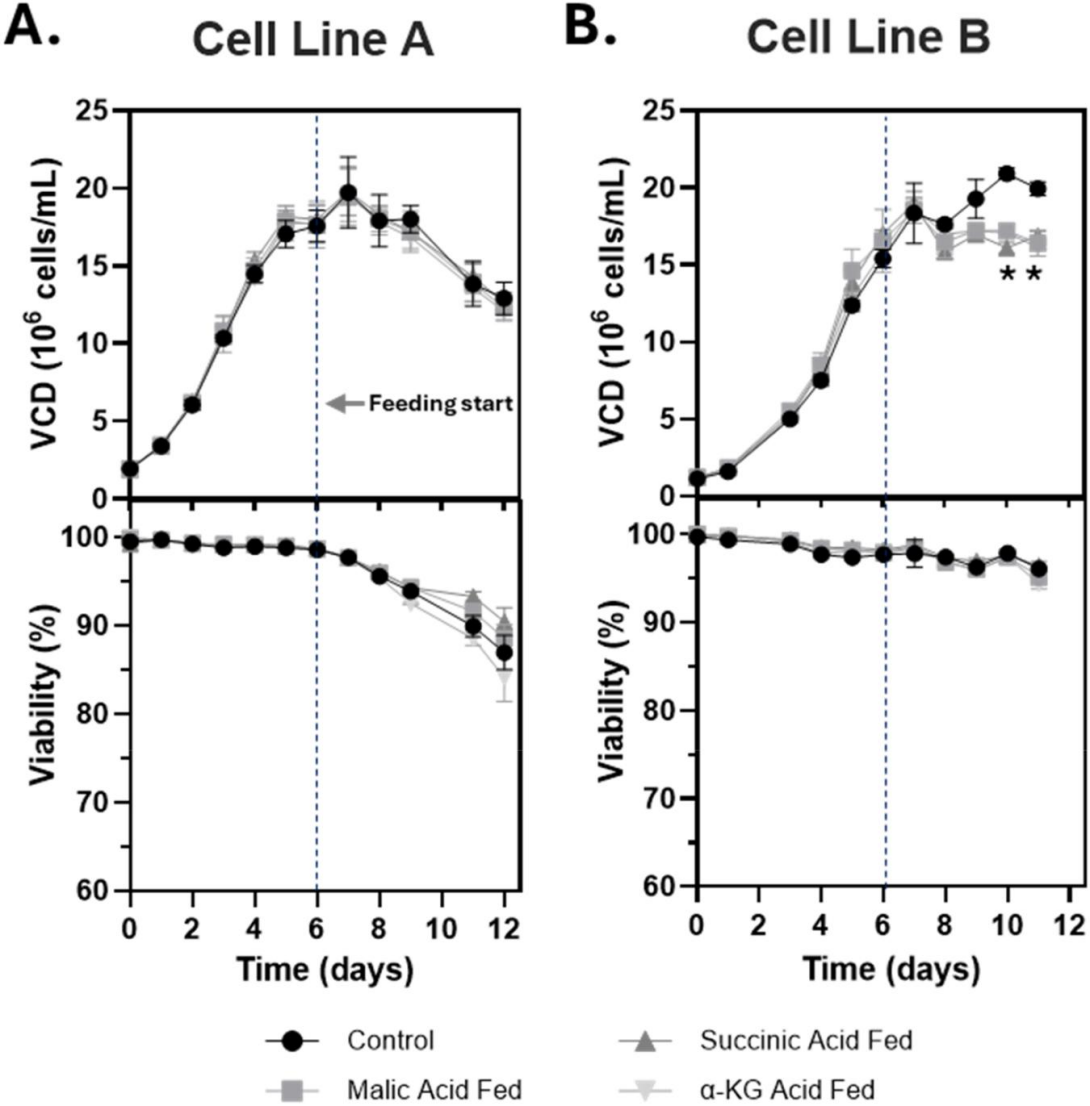
Cell culture growth under different TCA cycle intermediate feeding conditions. Αlpha‐KG acid, malic acid, or succinic acid was delivered to the bioreactors starting on day 6 automatically when pH was higher than 6.95. (A) VCD and viability for Cell line A. (B) VCD and viability for Cell line B. Mean ± SEM. *n* ≥ 2. *p < 0.05 for comparison of TCA cycle intermediate‐fed cultures to the control.

The effects of TCA cycle intermediate feeding on cell growth, viability, and qP differed between the two CHO cell lines, highlighting the complexity of cellular responses to varying supplementation strategies. In the first 6 days of culture, prior to TCA cycle intermediate feeding, VCD and viability were comparable across all conditions and between the two cell lines. However, after the introduction of TCA cycle intermediates for pH control on day 6, Cell Line A maintained consistent VCD across all conditions (Figure 1A), while Cell Line B exhibited a slight, yet significant, reduction in VCD from day 10 onward in response to TCA cycle intermediate feeding (Figure 1B). This finding suggests that TCA cycle intermediate supplementation negatively impacts the peak VCD of Cell Line B, likely due to cell‐specific sensitivity. Similar reductions in peak VCD, driven by cell line‐dependent responses to TCA cycle intermediate feeding, have been previously reported (Zhang et al. 2020; Yao et al. 2021; Saldanha et al. 2023). Cell viability exhibited clear divergence between the two cell lines following intermediate supplementation. In Cell Line A, viability declined to below 90% by the end of the 12‐day culture. In contrast, Cell Line B maintained viabilities above 95% across all conditions. Interestingly, the reduction in viability observed in Cell Line A was also present in control cultures, suggesting that the decline may be driven by cell‐specific intrinsic factors unrelated to TCA cycle intermediate feeding.

**Figure 2:**
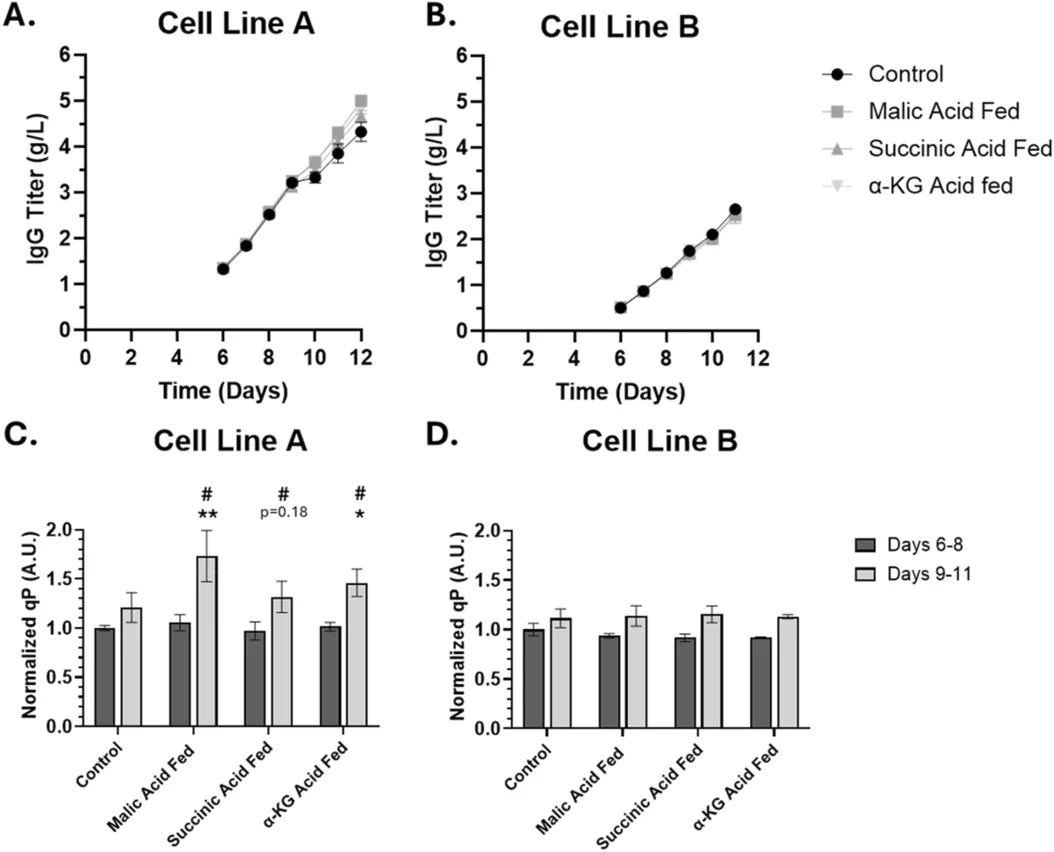
Effect of TCA cycle intermediate feeding on IgG titer and qP during early and late stationary phases. IgG titer profiles during fed‐batch culture following TCA cycle intermediate supplementation (succinic acid, malic acid, or α‐KG acid) starting on day 6 for (A) Cell Line A and (B) Cell Line B. Normalized qP values for (C) Cell Line A and (D) Cell Line B under control and TCA cycle intermediate‐fed conditions. Data are displayed for two time windows: days 6–8 (dark bars) and days 9–11 (light bars), corresponding to early and late stationary phases, respectively. Values are normalized to the control culture at days 6–8. Mean ± SEM. *n* ≥ 2. *p < 0.05, **p < 0.01 for comparison of normalized qP to the control culture at days 6–8. ^#^ *p* < 0.05 for comparison of normalized qP during late vs. early stationary phase under the same feeding condition.

Despite cell line‐specific differences in growth, volumetric IgG titers in Cell Line A were modestly higher under malic and α‐KG acid feeding relative to the control by late stationary phase (Figure 2A). In the control condition, the deviation from a linear titer increase between days 9 and 10 was associated with elevated measurement variability, as indicated by the larger error bars from day 9 to the end of the culture. In Cell Line B, volumetric titers remained comparable across conditions relative to the control, indicating minimal impact of intermediate feeding despite differences in VCD (Figure 2B). To determine whether these titer trends reflected changes in qP rather than differences in culture density alone, qP was evaluated over time following the introduction of TCA cycle intermediate feeding on day 6. During the first 2 days post‐feeding, normalized qP was similar across conditions for each cell line. By late stationary phase (days 9–11), Cell Line A showed a significant upward shift in qP under all intermediate‐fed conditions relative to early stationary phase (days 6–8), which exceeded the increase observed in control cultures (Figure 2C). Succinic acid feeding showed a higher normalized qP than the control, although the difference was not statistically significant. Thus, TCA cycle intermediate feeding resulted in consistent productivity improvements during late stationary phase following an initial adaptation period during early stationary phase. In contrast, Cell Line B exhibited insignificant changes in qP over time following intermediate feeding, which closely mirrored the control culture (Figure 2D). Taken together, the results indicate that TCA cycle intermediate feeding elicits delayed qP increases in Cell Line A, while Cell Line B exhibits no measurable improvement. These differences likely reflect distinct metabolic adaptations and differential utilization of supplemented intermediates by each cell line.

### 3.2 Glucose Consumption Was Reduced and, in Cell Line A, the Lactate Switch Was Enhanced by TCA Cycle Intermediate Supplementation

**Figure 3:**
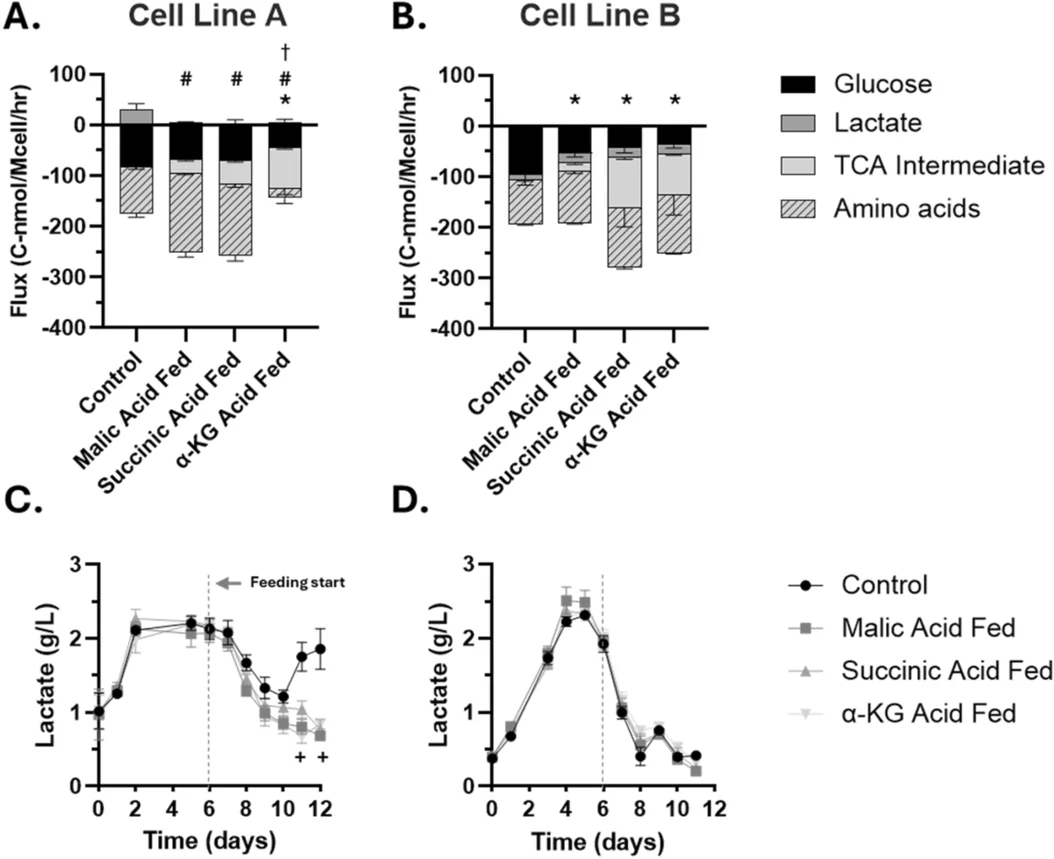
Uptake of key carbon sources during late stationary phase and extracellular lactate profiles in cultures over time. Net carbon contributions from nutrients supplied in the culture medium during days 10–12 for (A) Cell line A and (B) days 9–11 for Cell line B. Amino acid consumption and production rates for each cell line can be found in Supporting Information Figures S8 and S9. The consumption rate was expressed on a carbon‐moles basis by multiplying the measured rate by the number of carbon atoms in each molecule. A negative flux value indicates net consumption, while a positive flux value indicates net production. Extracellular lactate concentrations over time for (C) Cell Line A and (D) Cell Line B. Mean ± SEM. *n* ≥ 2. *p < 0.05 for comparison of glucose flux to the control. #*p* < 0.05 for comparison of lactate flux to the control. ^†^ *p* < 0.05 for comparison of total amino acid flux to the control. ^†^ *p* < 0.05 for comparison of lactate concentration in intermediate‐fed cultures to the control.

We next examined how TCA cycle intermediate feeding influences the uptake of key carbon sources, specifically glucose, lactate, and the supplemented TCA cycle intermediates. Consumption of TCA cycle intermediates was confirmed by measuring their levels in spent media, and substrate consumption rates were calculated on a carbon‐mole basis. During days 6–8, glucose consumption was comparable across conditions in both lines (Supporting Information Figure S3). However, significant differences in glucose consumption became apparent after day 8, further highlighting the delayed effects of TCA cycle intermediate feeding. In Cell Line A, glucose consumption was significantly lower in the α‐KG acid‐fed condition compared to the control, while malic acid and succinic acid‐fed conditions exhibited a trend toward reduced glucose consumption, although not statistically significant (Figure 3A). In Cell Line B, glucose consumption was significantly lower in all TCA cycle intermediate‐fed conditions compared to the control (Figure 3B).

The consumption of TCA cycle intermediates was also calculated on a carbon‐moles basis, confirming that these intermediates contribute significantly to the cellular carbon balance when supplied in the medium, as previously reported (Zhang et al. 2020; Chong et al. 2010). During the late stationary phase, the combined carbon uptake from glucose and supplemented succinic or α‐KG acid exceeded glucose‐derived carbon uptake in control cultures of both cell lines, whereas malic acid provided a smaller contribution to total carbon uptake. This finding indicates that TCA cycle intermediate feeding compensated, at least in part, for the significant reduction in glucose consumption observed under intermediate‐fed conditions. Moreover, lactate metabolism exhibited distinct patterns of consumption and production in the two cell lines. Cell Line A produced lactate under control conditions but shifted to lactate consumption following TCA cycle intermediate supplementation (Figure 3C). This shift toward increased lactate consumption during TCA cycle intermediate feeding, either with individual acids or a mixed cocktail, has been previously reported (Zhang et al. 2020; Saldanha et al. 2023). In contrast, Cell Line B consistently consumed lactate across all conditions (Figure 3D), suggesting a more robust lactate switch and an increased reliance on lactate oxidation to support energy generation during the stationary phase. Together, these results indicate that TCA cycle intermediate supplementation modulates carbon metabolism in both cell lines, promoting lactate utilization while reducing dependence on glucose as the primary carbon source.

**Figure 4:**
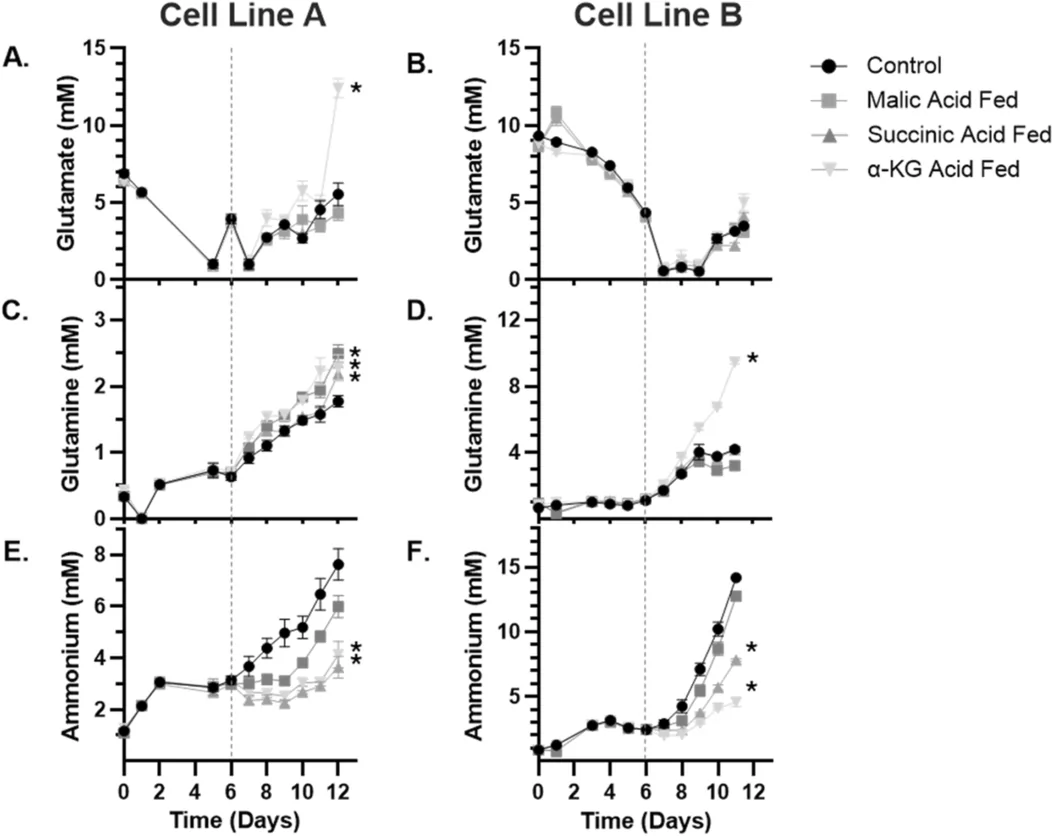
Extracellular metabolite profiles. Extracellular levels of (A and B) glutamate, (C and D) glutamine, and (E and F) ammonium in cultures of Cell Line A (left) and Cell Line B (right) over time. Glutamic acid was fed whenever extracellular glutamate concentrations dropped below 3 mM; the glutamate concentrations shown correspond to the values measured prior to any feeding event. Mean ± SEM. *n* ≥ 2. *p < 0.05 relative to the control at the final day of culture.

The net carbon contribution from amino acid uptake depended on both the cell line and feeding condition. In Cell Line A, feeding α‐KG acid significantly reduced net amino acid uptake, primarily due to elevated glutamate excretion (Figure 4A). In contrast, Cell Line B exhibited more consistent contributions from amino acids across all feeding conditions. As with lactate metabolism, differences in basal amino acid metabolism between the two cell lines may have led to variation in the response to TCA cycle intermediate feeding.

### 3.3 TCA Cycle Intermediate Feeding Alleviates Ammonium Accumulation and Affects Glutamine Metabolism in a Cell‐Specific Manner

Given the carbon flux changes under intermediate feeding, and the central role of α‐KG in transamination reactions, we next assessed nitrogen metabolism by measuring glutamate, glutamine, and ammonium extracellular concentrations. Relative to control, α‐KG acid feeding significantly increased extracellular glutamate in Cell Line A during late stationary phase (Figure 4A). However, this glutamate elevation was minimal in Cell Line B (Figure 4B). Intracellular measurements further indicated a larger glutamate pool in Cell Line A than in Cell Line B (Supporting Information Figure S4), suggesting that surplus glutamate was preferentially secreted rather than amidated to glutamine in Cell Line A (Figure 4C). In contrast, Cell Line B redirected excess carbon from α‐KG feeding toward glutamine, yielding higher extracellular glutamine concentrations compared to Cell Line A (Figure 4D).

Ammonium accumulation was markedly influenced by TCA cycle intermediate feeding in both cell lines (Figure 4E,F). In Cell Line A, control cultures exhibited a continuous rise in ammonium concentration after Day 6, whereas all intermediate‐fed conditions exhibited a delayed rise in ammonium concentration that reached a lower peak value. Among these, α‐KG acid and succinic acid feeding provided the strongest reductions, while malic acid feeding led to a moderate decline relative to control. This relationship suggests that enhanced conversion of α‐KG to glutamate and glutamine contributed to lower ammonium accumulation. In Cell Line B, similar patterns emerged, with control cultures again showing the greatest ammonium accumulation while α‐KG acid and succinic acid feeding resulted in pronounced reductions. However, their nitrogen redistribution differed from Cell Line A. α‐KG acid‐fed cultures exhibited the highest glutamine levels and lowest ammonium concentrations, consistent with active nitrogen assimilation into glutamine, whereas succinic acid‐fed cultures maintained low ammonium levels without a corresponding rise in glutamine. Collectively, these results reveal that TCA cycle intermediate supplementation not only alleviates ammonium accumulation but also rewires nitrogen metabolism in a substrate‐specific and cell line‐dependent manner.

### 3.4 ^13^C‐MFA Reveals Upregulated TCA Cycle Metabolism in Intermediate‐Fed Bioreactors

Next, we applied ^13^C‐MFA to cultures with or without TCA cycle intermediate feeding to investigate the effects on intracellular metabolism. Our findings demonstrate distinct impacts on central carbon fluxes, particularly in glycolysis and TCA cycle pathways, providing insight into the metabolic phenotype of each cell line under different feeding conditions.

**Figure 5:**
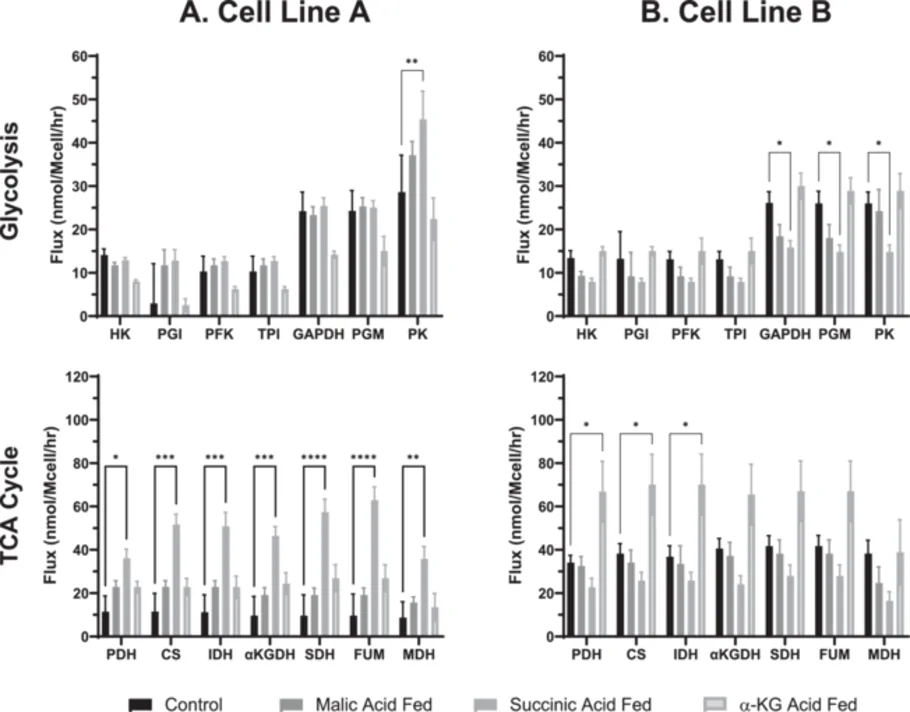
Metabolic flux reprogramming during late stationary phase due to TCA cycle intermediate feeding. Absolute intracellular fluxes for glycolysis (top panels) and the TCA cycle (bottom panels) are shown for (A) Cell Line A during the isotope labeling experiment (i.e., days 10–12) and (B) Cell Line B during the isotope labeling experiment (i.e., days 9–11) of fed‐batch culture, as determined by ^13^C‐MFA. Mean ± SEM. *p < 0.05, **p < 0.005, ***p < 0.0005, ****p < 0.00005 compared to control.

In Cell Line A, fluxes throughout upper glycolysis were similar in TCA cycle intermediate‐fed cultures and control cultures, with the exception of the α‐KG acid‐fed condition that showed reduced glycolytic flux (Figure 5A). Only the succinic acid‐fed cultures exhibited a significant increase in pyruvate kinase (PK) flux compared to control, which serves to balance the elevated cataplerotic flux from phosphoenolpyruvate carboxykinase (PEPCK, discussed further below). Consistently, most TCA cycle fluxes were significantly elevated in the succinic acid‐fed condition compared to the control. While malic acid and α‐KG acid also increased absolute TCA cycle fluxes, these changes were not statistically significant. In contrast, absolute glycolytic fluxes of Cell Line B were reduced in malic acid‐ and succinic acid‐fed cultures relative to α‐KG acid‐fed and control conditions (Figure 5B). Interestingly, TCA cycle fluxes in Cell Line B were consistently higher in α‐KG acid‐fed cultures compared to the control, further demonstrating the ability of TCA cycle intermediate feeding to enhance mitochondrial energy metabolism. Notably, no significant differences were observed between the HCl acid and control conditions for Cell Line B (Supporting Information Figure S5), ruling out the contribution of altered CO_2_ availability to changes in glycolytic or TCA cycle fluxes.

**Figure 6:**
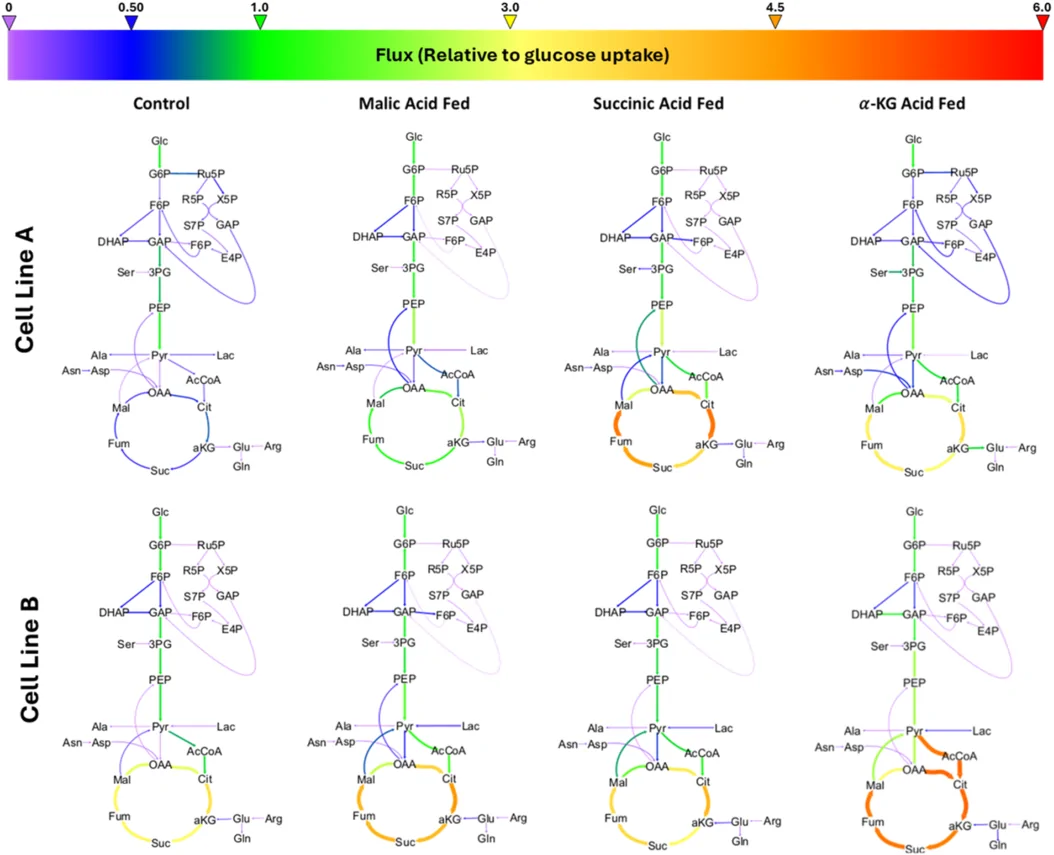
Flux re‐routing during late stationary phase due to TCA cycle intermediate feeding. ^13^C‐MFA was used to generate flux maps for Cell Line A (top row) and Cell Line B (bottom row) under control and TCA cycle intermediate‐fed conditions (malic acid, succinic acid, and α‐KG acid). Net fluxes are shown on a carbon‐moles basis, normalized to glucose uptake during the ^13^C labeling period and color‐coded according to relative magnitude (scale shown above). Arrow thickness is proportional to flux magnitude and follows the same scale as the color coding. These flux maps highlight cell line‐specific responses and altered carbon partitioning driven by intermediate supplementation. *n* ≥ 2.

It is important to note that many of the differences in absolute fluxes correspond to variations in the glucose uptake rate across conditions. To assess the effects of TCA cycle intermediate feeding on the intracellular distribution of carbon fluxes while controlling for differences in glucose uptake, we converted all absolute fluxes to relative fluxes by normalizing to the glucose uptake rate determined under each condition (Supporting Information Figures S6 and S7). Upon normalization, certain differences between the feeding conditions become more apparent (Figure 6). In glycolysis, all three TCA cycle intermediate‐fed conditions for Cell Line A exhibited a trend toward increased relative PK flux. Among these, malic acid and succinic acid supplementation led to statistically significant increases in relative PK flux compared to the control (Supporting Information Figure S6A). This enhanced activity can be attributed to increased pyruvate cycling via the sequential steps of PK, pyruvate carboxylase (PC), and PEPCK (Supporting Information Figure S6D). Pyruvate cycling is an ATP‐consuming process that is often upregulated when cells are in a high‐energy state or when there are imbalances between oxidative and anaplerotic substrates entering the TCA cycle (Bednarski et al. 2024).

After normalizing to glucose uptake, relative TCA cycle fluxes were significantly enhanced in cultures fed α‐KG acid or succinic acid compared to control cultures of Cell Line A (Figure 6). Malic acid‐fed conditions also displayed moderate increases in flux, though not statistically significant (Supporting Information Figure S6C). Succinic acid‐fed cultures of Cell Line A exhibited the highest relative TCA cycle fluxes, aligning with elevated intracellular succinate levels, which were 5–6 times higher than in control cultures (Supporting Information Figure S4). Specifically, all relative TCA cycle fluxes were significantly elevated in succinic acid‐fed cultures except for malate dehydrogenase (MDH), which showed increases that were not statistically significant due to partial re‐routing of flux through malic enzyme (ME) (Supporting Information Figure S6D). In addition to ME activation, α‐KG acid‐fed cultures of Cell Line A exhibited a partial diversion of α‐KG toward glutamate synthesis, as indicated by elevated intracellular accumulation that persisted over time (Supporting Information Figure S4) and increased glutamate secretion (Figure 4A). This diversion slightly reduced the flux of α‐KG through the TCA cycle, explaining why α‐KG acid feeding did not achieve the same extent of TCA cycle flux enhancement (Figure 6) or elevation of downstream TCA cycle metabolites (Supporting Information Figure S4) compared to succinic acid feeding. Notably, Cell Line B displayed substantially higher relative TCA cycle fluxes under control conditions than Cell Line A, which minimized the relative effect of intermediate feeding. This elevated TCA cycle activity can be partly attributed to higher consumption of lactate (Figure 3), thereby providing an additional carbon source that sustains oxidative metabolism even without external intermediate supplementation.

**Figure 7:**
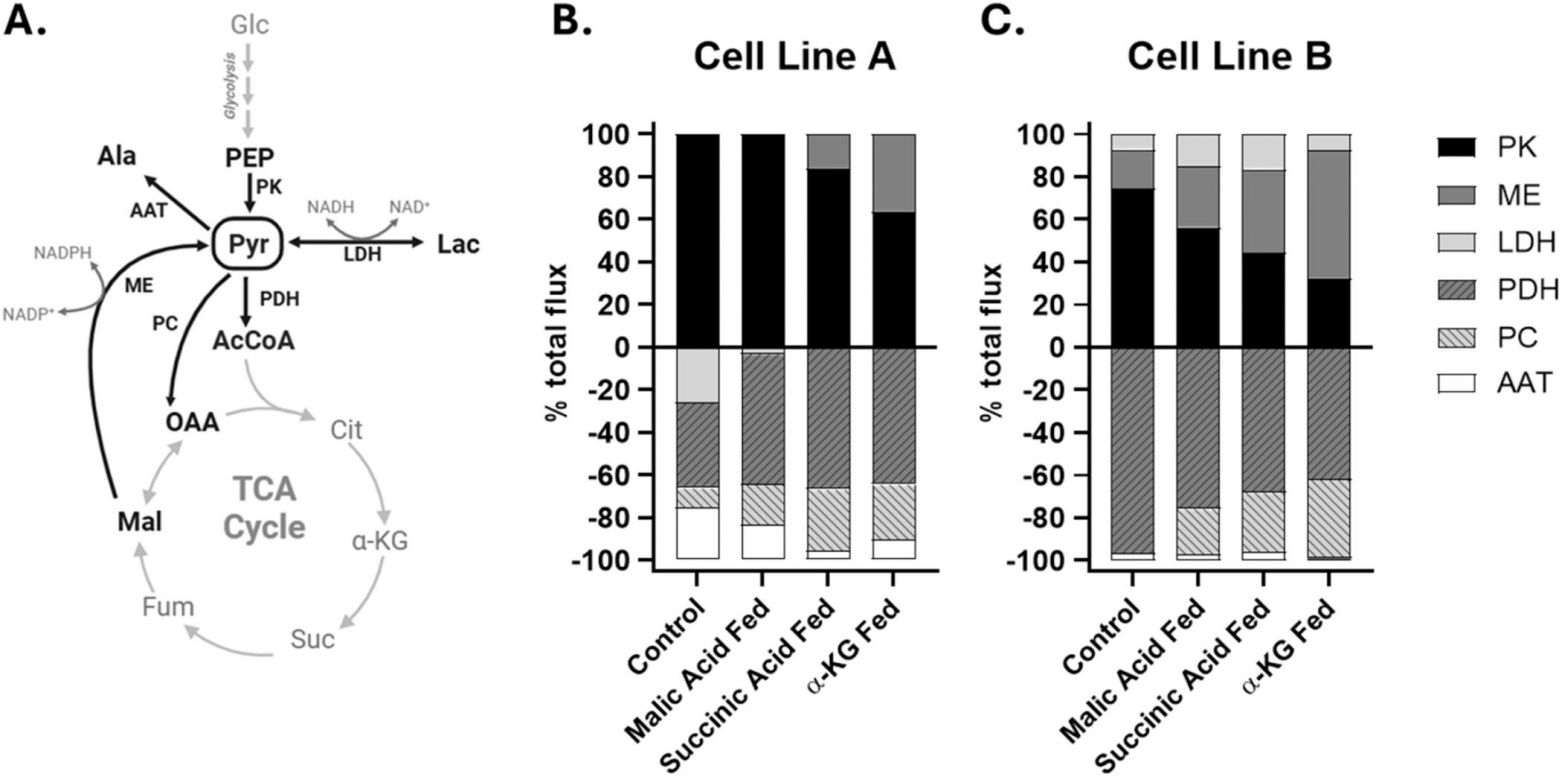
Flux repartitioning at the pyruvate node under TCA cycle intermediate feeding. (A) Schematic representation of fluxes that produce and consume pyruvate. Metabolic flux contributions (in percentages) from reactions that produce (positive %) and consume (negative %) pyruvate in (B) Cell Line A and (C) Cell Line B under different feeding conditions.

Interestingly, feeding of TCA cycle intermediates repartitioned pyruvate‐producing fluxes, with a notable decrease in PK contribution in both cell lines (Figure 7). This suggests that the metabolic demand for pyruvate was increasingly met through ME rather than PK when TCA cycle intermediates were supplemented. The increase in ME flux was partially balanced by enhanced anaplerotic flux of pyruvate returning to the TCA cycle via PC. In Cell Line B, this flux circuit creates a futile cycle involving ME, PC, and MDH. In Cell Line A, PDH and PC fluxes were concurrently upregulated, which led to a more robust increase in TCA cycle fluxes by promoting complete oxidation of the supplemented TCA cycle intermediates. Differences in pyruvate flux partitioning through PDH between the two cell lines are possibly influenced by differences in their lactate metabolism. In Cell Line B, where lactate was consumed under all conditions, the reliance on PDH flux decreased when TCA cycle intermediates were fed. Conversely, in Cell Line A, the reliance on PDH flux was higher in the TCA cycle intermediate‐fed cultures, likely due to their decreased lactate production compared to control cultures.

### 3.5 TCA Cycle Intermediate Feeding Rewires Cytosolic Redox Metabolism

**Figure 8:**
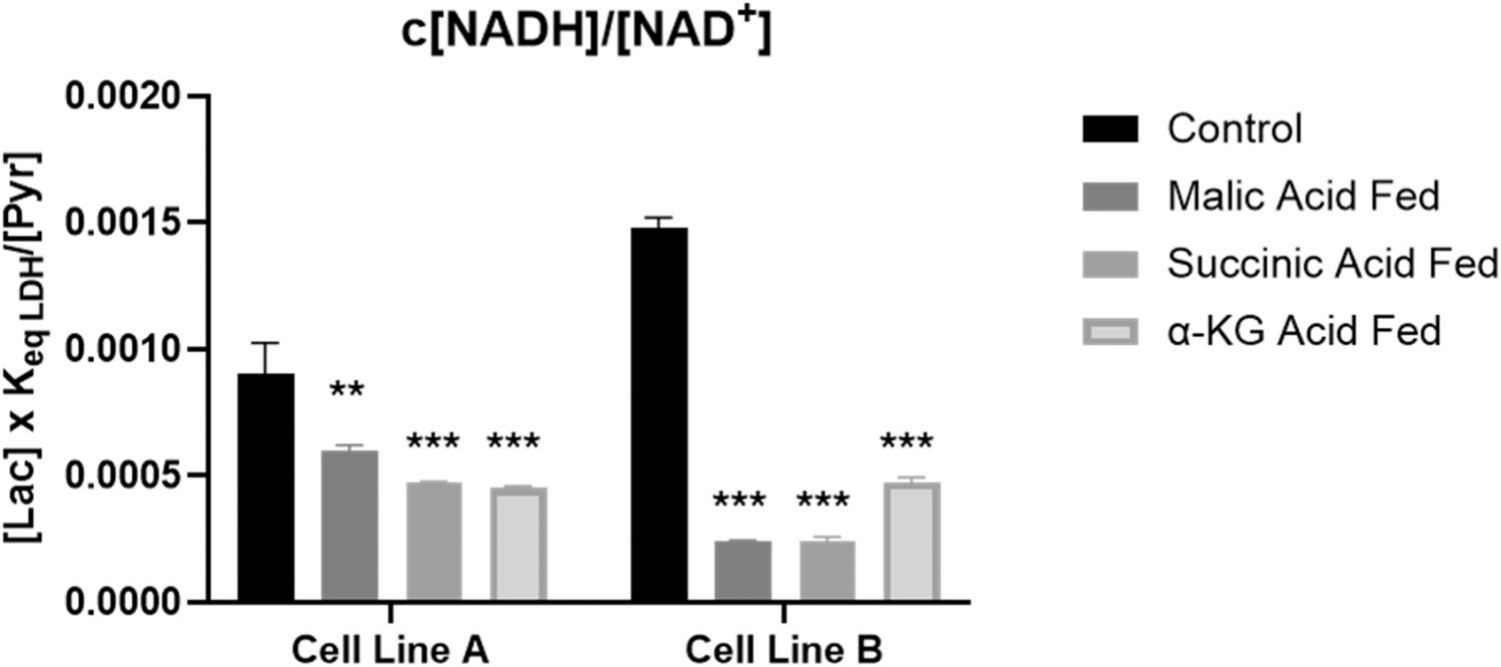
Cytosolic NADH/NAD^+^ redox state in Cell Line A and Cell Line B on day 11 of culture. Ratios were estimated based on the near‐equilibrium assumption of lactate dehydrogenase (LDH), using intracellular concentrations of lactate and pyruvate as described in Section 2.7. Mean ± SEM for each condition (*n* ≥ 2). *p < 0.05, **p < 0.005, ***p < 0.0005 vs. control.

To assess cytosolic redox balance, we estimated the cytosolic NADH/NAD^+^ ratio during late stationary phase. Across all intermediate‐fed conditions, the NADH/NAD^+^ ratio was significantly lower than in control cultures of both cell lines (Figure 8). Absolute ratios differed by cell line: under control conditions, Cell Line B exhibited a higher NADH/NAD^+^ ratio than Cell Line A, consistent with its sustained lactate consumption phenotype. Nevertheless, the direction of the redox shift in response to intermediate feeding was the same in both lines (toward a more oxidized cytosol), with a larger fold‐change in Cell Line B.

## 4 Discussion

Modulating central carbon metabolism through metabolic engineering or media optimization is a longstanding strategy for enhancing recombinant protein production in CHO cells (Fischer et al. 2015; Sacco et al. 2023, 2022; Combe and Sokolenko 2021). Among these, supplementation with TCA cycle intermediates has shown promise for reducing lactate and ammonium accumulation and supporting oxidative metabolism (Zhang et al. 2020; Saldanha et al. 2023). A previous study demonstrated that feeding these intermediates in acidic form can serve a dual role: stabilizing culture pH while supplying anaplerotic carbon (Zhang et al. 2020). Building on that framework, this study used ^13^C‐MFA to uncover how acidic TCA cycle intermediate feeding rewires central carbon metabolism, redox balance, and recombinant protein production capacity in two industrial CHO cell lines.

Despite no effect on growth or viability during exponential phase (Figure 1), TCA cycle intermediate feeding enhanced qP during late stationary phase for Cell Line A but not Cell Line B (Figure 2). These findings align with prior reports that qP gains from TCA cycle intermediate supplementation are dependent on compound selection, dosing, and timing of addition (Zhang et al. 2020; Yao et al. 2021; Saldanha et al. 2023). In particular, studies by Zhang et al. (2020) and Yao et al. (2021) showed productivity enhancements only when feeding occurred during the stationary phase at optimized concentrations. In our study, the intermediates were delivered according to pH buffering needs, not a fixed metabolite concentration, introducing variation in the effective dose. Differences in the quantity of acid delivered across cultures (see Supporting Information Table S2) could partly explain the lack of consistency with prior studies and also variation between different feeds in our study. These findings reinforce the hypothesis that TCA cycle intermediate supplementation supports biosynthetic metabolism via a time‐dependent process through which central carbon and energy metabolism gradually adapts to support increased productivity (Saldanha et al. 2023).

Another key insight from this study was the reduction in glucose consumption across TCA cycle intermediate‐fed cultures (Figure 3), accompanied by increased reliance on alternative carbon sources and distinct lactate dynamics that paralleled the changes in qP shown in Figure 2. In Cell Line A, TCA cycle intermediate feeding induced a clear lactate switch, from production to consumption, which coincided with increased qP. These observations are consistent with prior reports that lactate consumption can reflect reduced glycolytic overflow (Saldanha et al. 2023) and increased mitochondrial oxidation (Sacco et al. 2023; Zhang et al. 2020; Templeton et al. 2013; Saldanha et al. 2023), conditions often associated with increased recombinant protein productivity. In contrast, Cell Line B exhibited a lactate‐consuming phenotype even under control conditions. As a result, intermediate feeding did not induce a metabolic shift comparable to that seen in Cell Line A. Although glucose uptake decreased significantly in the intermediate‐fed conditions, lactate consumption remained similar across all conditions, and no significant improvements in qP were observed. These results imply that the productivity benefit of intermediate feeding depends on induction of lactate shift, coupled with repartitioning of pyruvate flux into the TCA cycle via PDH (Figure 7).

Brunner et al. (2017) previously reported that elevated pCO_2_ (150 mmHg) can inhibit the lactate shift in CHO batch and fed‐batch cultures when compared to control cultures maintained at lower pCO_2_ levels (< 100 mmHg) and linked this effect to altered intracellular redox balance and reduced oxidative capacity. Thus, differences in pCO_2_ may contribute to changes in lactate metabolism and broader oxidative metabolism. After day 6 in our study, when CO_2_‐based acidification was replaced with TCA cycle intermediate supplementation, pCO_2_ trended lower in all intermediate‐fed cultures (Supporting Information Figure S1C,D). However, the lactate shift was not governed solely by CO_2_ sparging, since both cell lines experienced lower pCO_2_ under intermediate feeding but differed in their lactate phenotypes. Cell Line A underwent a complete switch from lactate production to lactate consumption during stationary phase, whereas Cell Line B already exhibited a lactate shift under control conditions and did not exhibit a change in its lactate profile due to intermediate supplementation. These findings suggest that reduced pCO_2_ may contribute to lactate regulation, but that the response to TCA cycle intermediate feeding depends on cell‐line‐specific characteristics that regulate metabolic state and carbon flux distribution. Among the tested intermediates, malic acid was consumed most slowly by both cell lines. This trend suggests a transport or utilization bottleneck specific to malate, consistent with prior studies reporting delayed malate uptake during the stationary phase (Chrysanthopoulos et al. 2010; Chong et al. 2009; Aranibar et al. 2011; Miccheli et al. 2006; Chong et al. 2010). Interestingly, a study wherein fumarate was supplemented to CHO cell cultures reported even greater malate accumulation in the culture supernatant than when malate itself was fed (Saldanha et al. 2023), pointing to a potential intracellular metabolic bottleneck or feedback inhibition. Notably, overexpression of mitochondrial MDH has been shown to improve malate assimilation (Chong et al. 2010), supporting the hypothesis that downstream metabolic processing, rather than external availability, is rate‐limiting. Despite the lower malate uptake, net carbon influx from all sources was typically higher in intermediate‐fed cultures than in controls (Figure 3A,B), indicating a metabolic shift toward increased utilization of organic acids and reduced reliance on glucose. Intermediate feeding also lowered the cytosolic NADH/NAD^+^ ratio in both cell lines (Figure 8), consistent with reduced NADH production from glycolysis. In Cell Line A, this redox shift likely contributed to enhancing the lactate switch and redirecting pyruvate toward PDH (Figure 7).

In addition to modulating central carbon metabolism, feeding TCA cycle intermediates notably influenced nitrogen metabolism in a cell–specific manner. In Cell Line A, α‐KG acid feeding reduced ammonium accumulation by promoting substantial glutamate production, while succinic acid feeding had a similar effect on ammonium but without stimulating glutamate production (Figure 4, left panel). The latter response could possibly be attributed to simultaneous reductions in asparagine and serine uptake along with increased glutamine production (Supporting Information Figure S8). In contrast, Cell Line B exhibited a significant increase in glutamine production under α‐KG acid feeding without a corresponding rise in glutamate, reflecting more efficient conversion of glutamate to glutamine by the glutamine synthetase enzyme (Figure 4, right panel). These observations aligned with previous studies showing that the metabolic outcome of α‐KG supplementation depends strongly on the intrinsic nitrogen‐handling capacity of the cell line (Ha and Lee 2014). Succinic acid feeding also reduced ammonium production by Cell Line B, but unlike in Cell Line A, this effect was most strongly associated with elevated conversion of pyruvate to alanine (Supporting Information Figure S9). It is also interesting to note that the basal amino acid metabolism of each cell line is quite distinct, with Cell Line A preferentially consuming asparagine and producing both aspartate and glutamate while Cell Line B exhibits balanced consumption of asparagine, aspartate, and glutamate under control conditions. These differences highlight the need to consider cell line‐specific effects on nitrogen balance when optimizing feeding strategies for bioprocess performance.

^13^C‐MFA revealed that supplementation with TCA cycle intermediates differentially modulates central carbon fluxes depending on the compound and cell line (Figure 5). In Cell Line A, succinic acid feeding elevated absolute TCA cycle fluxes, while malic and α‐KG acid had more subtle effects. In contrast, Cell Line B exhibited the strongest TCA cycle flux enhancement under α‐KG supplementation, with succinic and malic acid providing no boost in TCA cycle flux. In both cell lines, malic acid had the smallest impact on carbon flux, consistent with its slower uptake (Figure 3) and limited incorporation into the TCA cycle (Figure 6) compared to α‐KG or succinic acid. The diminished response to malic acid feeding could also reflect the more diverse downstream fates of malate—it can be directly converted to pyruvate (via malic enzyme) or fumarate (via fumarase), and it can be routed through MDH to either aspartate or citrate—which can serve to effectively assimilate the added carbon source without significant TCA flux perturbations.

Differences in the response to TCA cycle intermediate feeding between the two cell lines can be attributed to differences in their basal metabolism, as well as the unique routes through which they metabolize the supplemented carbon sources. First, the overall higher magnitude of oxidative fluxes in Cell Line B compared to Cell Line A, which stems primarily from elevated lactate consumption, reduced the dependence of Cell Line B on exogenous TCA cycle intermediates and limited the extent of flux re‐routing in response to intermediate feeding (Figure 6). Second, succinic acid feeding stimulated PEPCK and PK flux in Cell Line A but inhibited glycolysis in Cell Line B, while α‐KG acid feeding reduced glycolysis in Cell Line A but not in Cell Line B (Figure 5). Third, differences in amino acid metabolism could underlie the unique response of each cell line to TCA cycle intermediate feeding. For example, α‐KG acid feeding led to substantial glutamate production and aspartate consumption in Cell Line A (Supporting Information Figure S8), while most of the supplemented α‐KG was routed through the TCA cycle in Cell Line B (Figure 6), with glutamine as the major overflow product (Figure 4D and Supporting Information Figure S9).

Importantly, while increased TCA cycle flux has been previously correlated with higher qP in CHO cells (Templeton et al. 2017a; Sacco et al. 2023; Zhang et al. 2020; Templeton et al. 2013), our results suggest that this relationship is not always directly proportional. In our study, significant increases in TCA cycle flux, particularly under α‐KG‐ and succinic acid‐feeding, did not consistently translate into statistically significant improvements in qP. Nevertheless, modest increases in qP were still observed, supporting a model in which enhanced oxidative metabolism contributes to, but does not solely dictate, productivity gains. Proteomic studies further support this observation. Strasser et al. (2021). reported that high‐producing CHO clones exhibit an activated TCA cycle and elevated oxidative phosphorylation, suggesting that higher energy availability can support increased protein synthesis. However, boosting central carbon metabolism does not always raise qP. Wilkens et al. (2015) overexpressed MDH II to increase TCA cycle flux, but IgG‐producing CHO pools did not improve qP and sometimes showed lower titer and qP, indicating that higher metabolic efficiency does not always correspond to increased productivity. These apparent discrepancies emphasize the multifactorial control of metabolic flux and IgG productivity in CHO cells.

While our data reveal clear metabolic responses to TCA cycle intermediate feeding, several features of the experimental design constrain how broadly these findings should be interpreted. Because intermediates were delivered primarily as pH control agents, the dosing profile was set by culture‐specific acid/base demand rather than a defined bolus or fixed supplementation schedule. This approach shares similarities with other pH‐coupled feeding strategies, such as high‐end pH‐controlled glucose delivery, which can reduce lactate accumulation and improve culture performance by regulating nutrient availability (Gagnon et al. 2011). However, TCA cycle intermediates directly contribute to culture acidification while simultaneously supplying anaplerotic carbon to fuel mitochondrial metabolism. This differs from pH‐controlled glucose delivery, in which effects on pH are mediated indirectly by regulating glucose availability and lactate metabolism rather than by direct acid supplementation.

Key parameters, including the pK_a_ of each selected intermediate, its buffering capacity at the culture pH, and the cumulative amount fed were not explicitly optimized in this study. These factors could meaningfully influence both effective delivery and cellular response and therefore warrant further investigation in future work. In addition, the ^13^C‐MFA results capture stationary phase metabolism during two discrete windows, which may not resolve transient adaptations that occur after feeding begins, particularly when the number of biological replicates is limited. Likewise, although the LDH equilibrium relationship offers a practical redox proxy, it does not directly report compartment‐specific NADH/NAD^+^ pools or mitochondrial redox dynamics, which will require direct measurements of energy and redox markers to strengthen mechanistic interpretation. Finally, because this work interrogates stationary phase metabolism within a single fed‐batch platform and pH control strategy, the generality of the conclusions across process modes and feeding strategies remains to be fully established. For example, controlled dosing of each intermediate, alternative feeding regimens (e.g., mixed acids), and evaluation across broader operating conditions will be needed to more directly test causal links between TCA cycle flux rewiring and qP. However, any nutrient supplementation strategy must explicitly account for counterion load and osmolality shifts, since elevated osmolality can inhibit growth and alter product quality attributes (Dorai et al. 2009; Hassell et al. 1991; Pacis et al. 2011), complicating interpretation and potentially reshaping overall bioprocess performance in mAb‐producing CHO cell lines.

## 5 Conclusions

Integrating acidic TCA cycle intermediates into CHO fed‐batch operations as both pH‐control agents and metabolic supplements consistently rewired central carbon metabolism in a cell line‐ and compound‐specific manner. Across conditions, ^13^C‐MFA revealed a coordinated shift toward enhanced mitochondrial pyruvate oxidation, a more oxidized cytosolic redox state, and net lactate consumption, without impairing early growth or viability. These changes coincided with modest increases in qP, with the strongest response observed with malic acid feeding of Cell Line A. The magnitude of response depended on the host's intrinsic metabolic characteristics—particularly its capacity for lactate oxidation and nitrogen assimilation, indicating that a single “universal” feeding strategy is unlikely to be optimal. Collectively, our results support a physiological mechanism in which exogenous TCA cycle intermediates modulate metabolic state rather than acting as direct productivity effectors, thereby creating a cellular milieu favoring recombinant protein synthesis. Prior reports further suggest that such supplementation can increase mAb titer without adversely affecting critical quality attributes, supporting compatibility with industrial practice (Saldanha et al. 2023). These findings provide a framework for next‐generation pH‐control strategies that use acidic compounds with a dual function: controlling pH and simultaneously acting as metabolic substrates. These strategies can help to promote TCA cycle flux, enhance the lactate switch, and improve IgG productivity, as demonstrated in this study.

## Nomenclature

**Metabolites**

aKG: α‐ketoglutarate
AcCoA: acetyl‐coenzyme A
Ala: alanine
Arg: arginine
Asn: asparagine
Asp: aspartate
Cit: citrate
DHAP: dihydroxyacetone phosphate
E4P: erythrose‐4‐phosphate
F6P: fructose‐6‐phosphate
Fum: fumarate
G6P: glucose‐6‐phosphate
GAP: glyceraldehyde‐3‐phosphate
Glc: glucose
Gln: glutamine
Glu: glutamate
HCl: hydrochloric acid
Lac: lactate
Mal: malate
OAA: oxaloacetate
R5P: ribose‐5‐phosphate
Ru5P: ribulose‐5‐phosphate
S7P: sedoheptulose‐7‐phosphate
Ser: serine
Suc: succinate
X5P: xylulose‐5‐phosphate

**Enzymes**

aKGDH: α‐ketoglutarate dehydrogenase
AAT: alanine aminotransferase
CS: citrate synthase
FUM: fumarase
GAPDH: glyceraldehyde‐3‐phosphate dehydrogenase
GS: glutamine synthetase
HK: hexokinase
IDH: isocitrate dehydrogenase
LDH: lactate dehydrogenase
MDH: malate dehydrogenase
ME: malic enzyme
PC: pyruvate carboxylase
PDH: pyruvate dehydrogenase
PEP: phosphoenolpyruvate
PGI: phosphoglucose isomerase
PGM: phosphoglycerate mutase
PK: pyruvate kinase
SDH: succinate dehydrogenase
TPI: triose phosphate isomerase

## Conflicts of Interest

The authors declare no conflicts of interest.

## Supporting information


**Figure S1:** Offline pH and pCO_2_ profiles following replacement of CO_2_ sparging with TCA cycle intermediates on day 6. Offline pH profiles from day 6 to the end of culture are shown for (A) Cell Line A and (B) Cell Line B. pCO_2_ profiles over the same culture period are shown for (C) Cell Line A and (D) Cell Line B. Green dashed lines indicate the online pH‐control setpoint, and red dashed lines indicate the upper and lower pH‐control deadbands. Mean ± SEM, n = 2.**Figure S2:** Glucose profiles of the control and TCA cycle intermediate‐fed conditions over the fed‐batch study for (A) Cell Line A and (B) Cell Line B. Mean ± SEM. n = 2.**Figure S3:** Consumption of glucose during the first two days (6–8) of TCA cycle intermediate feeding in (A) Cell Line A and (B) Cell Line B. Mean ± SEM. n = 2.**Figure S4:** Intracellular metabolite concentrations during the 13C‐labeling study for Cell Line A and Cell Line B.**Figure S5:** Absolute fluxes of control and HCl acid conditions during days 10‒12 for Cell Line B.**Figure S6:** Cell line A fluxes normalized to glucose uptake during days 10–12.**Figure S7:** Cell line B fluxes normalized to glucose uptake during days 9–11.**Figure S8:** Amino acid consumption and production rates of Cell line A during the day 10–12 time period.**Figure S9:** Amino acid consumption and production rates of Cell line B during the day 9–11 time period.**Table S1:** Sum of squared residuals (SSR) for 13C MFA best‐fit solutions.**Table S2:** Average glutamic acid, TCA cycle intermediate, and glucose feeding profiles.

## Data Availability

The data that support the findings of this study are available from the corresponding author upon reasonable request.
