# Supplementary material for: Metabolic Flux Analysis Reveals Cell Line‐Specific Rewiring in CHO Cells Following TCA Cycle Intermediate Feeding for Bioprocess pH Control
Source: Biotechnol Bioeng. 2026 Jun 28;123(10):2713–28. doi: 10.1002/bit.70287 (PMC13576813; doi:10.1002/bit.70287)
Supplement: Supplementary file 1 [file BIT-123-2713-s001.docx]

**Supplementary Material for**

**Metabolic Flux Analysis Reveals Cell Line-Specific Rewiring in CHO Cells Following TCA Cycle**

**Intermediate Feeding for Bioprocess pH Control**

Sarah A. Sacco, Kevin J. Ruiz-Márquez, Rachel Moen, Irina Trenary, Xiaolin Zhang, Xiaowen Wang,

Malik Padellan, Henry Lin, Jamey D. Young

**Supplementary Tables**

**Supplementary Table S1. Sum of squared residuals (SSR) for ^13^C MFA best-fit solutions**.

| **Cell Line** | **Condition** | **Sum of Squared**  **Residuals (SSR)** | **LB** | **UB** | **DOF** |
| --- | --- | --- | --- | --- | --- |
| **A** | **Control** | 113.7 | 104.7 | 169.2 | 133 |
|  | **Malic acid Fed** | 145.6 | 88.1 | 147.7 | 116 |
|  | **Succinic acid Fed** | 166 | 103 | 166.8 | 133 |
|  | ***a*-KG acid Fed** | 121 | 82 | 139.8 | 109 |
| **B** | **Control** | 112.8 | 69.9 | 123.9 | 95 |
|  | **HCl acid Fed** | 111.7 | 75.7 | 130.7 | 101 |
|  | **Malic acid Fed** | 127.8 | 87.2 | 146.6 | 115 |
|  | **Succinic Acid Fed** | 105.3 | 70.8 | 125 | 96 |
|  | **α-KG Acid Fed** | 112 | 85.5 | 144.3 | 113 |

- Confidence intervals are based on a chi-square distribution with the indicated degrees of freedom (DOF). If the SSR is below the UB threshold, the fit is acceptable.
- LB = lower bound of expected SSR range, UB = upper bound of expected SSR range

**Supplementary Table S2. Average glutamic acid, TCA cycle intermediate, and glucose feeding profiles**

| **Cell line**  **ID** | **Condition** | **Total glutamic acid feed (mM) ^a^** | **Total glucose feed (g/L) ^a^** | **Total TCA cycle intermediate feed (mM) ^a^** |
| --- | --- | --- | --- | --- |
| **A** | **Control** | 11.4 | 29.5 | 0 |
|  | **α-KG acid** | 6.7 | 27.2 | 37.7 |
|  | **Malic acid** | 10.2 | 29.1 | 25.0 |
|  | **Succinic acid** | 10.5 | 29.7 | 32.3 |
| **B** | **Control** | 15.6 | 44.7 | 0 |
|  | **α-KG acid** | 13.3 | 41.5 | 31.5 |
|  | **Malic acid** | 13.7 | 41.6 | 36.5 |
|  | **Succinic acid** | 11.5 | 39.3 | 36.8 |

a. total glutamic acid, glucose and TCA cycle intermediates fed over 12 days (A) and 11 days (B).

**Supplementary Figures**

**Supplementary Figure S1**. Offline pH and pCO₂ profiles following replacement of CO₂ sparging with TCA cycle intermediates on day 6. Offline pH profiles from day 6 to the end of culture are shown for (A) Cell Line A and (B) Cell Line B. pCO₂ profiles over the same culture period are shown for (C) Cell Line A and (D) Cell Line B. Green dashed lines indicate the online pH-control setpoint, and red dashed lines indicate the upper and lower pH-control deadbands. Mean ± SEM, n = 2.

**Supplementary Figure S2**. Glucose profiles of the control and TCA cycle intermediate-fed conditions over the fed-batch study for (A) Cell Line A and (B) Cell Line B. Mean ± SEM. n = 2.

**Supplementary Figure S3**. Consumption of glucose during the first two days (6-8) of TCA cycle intermediate feeding in (A) Cell Line A and (B) Cell Line B. Mean ± SEM. n = 2.

**Supplementary Figure S4. Intracellular metabolite concentrations during the ^13^C-labeling study for Cell Line A and Cell Line B.** Intracellular levels of glutamate, succinate, aspartate, and malate were measured at multiple time points during fed-batch culture under control, malic acid-fed, succinic acid-fed, and α-KG acid-fed conditions. Time zero (T = 0 days) marks the initiation of the ^13^C-labeling experiment for Cell Line A (top panel) and Cell Line B (bottom panel). Mean ± SEM. n = 2.

**Supplementary Figure S5: Absolute fluxes of control and HCl acid conditions during days 10-12 for Cell Line B**. The absolute flux rates determined by ^13^C-MFA for reactions in (A) glycolysis, (B) PPP, (C) TCA cycle, and (D) other selected fluxes are shown. Mean ± SEM. n = 2.

**Supplementary Figure S6**: **Cell line A fluxes normalized to glucose uptake during days 10-12**. The flux rates determined by ^13^C MFA normalized to the glucose uptake rate for reactions in (A) glycolysis, (B) PPP, (C) TCA cycle, and (D) other selected fluxes are shown. Mean ± SEM. ***p* < 0.005, ****p* < 0.0005, *****p* < 0.00005 relative to the control.

**Supplementary Figure S7**: **Cell line B fluxes normalized to glucose uptake during days 9-11**. The flux rates determined by ^13^C MFA normalized to the glucose uptake rate for reactions in (A) glycolysis, (B) PPP, (C) TCA cycle, and (D) other selected fluxes are shown. Mean ± SEM. ***p* < 0.005, ****p* < 0.0005, *****p* < 0.00005 relative to the control.

**Supplementary Figure S8: Amino acid consumption and production rates of Cell line A during the day 10-12 time period**. A negative flux value indicates net consumption, while a positive value indicates net production. Mean ± SEM. **p <* 0*.*05 relative to the control.

**Supplementary Figure S9: Amino acid consumption and production rates of Cell line B during the day 9-11 time period**. A negative flux value indicates net consumption, while a positive value indicates net production. Mean ± SEM. **p <* 0*.*05 relative to the control.
